# Hirayama Disease: A Rare Disease with Unusual Features

**DOI:** 10.1155/2016/5839761

**Published:** 2016-12-21

**Authors:** S. Anuradha, Vanlalmalsawmdawngliana Fanai

**Affiliations:** Department of Medicine, Maulana Azad Medical College and Associated Hospitals, New Delhi 110002, India

## Abstract

Hirayama disease, also known as monomelic amyotrophy (MMA), is a rare cervical myelopathy that manifests itself as a self-limited, asymmetrical, slowly progressive atrophic weakness of the forearms and hands predominantly in young males. The forward displacement of the posterior dura of the lower cervical dural canal during neck flexion has been postulated to lead to lower cervical cord atrophy with asymmetric flattening. We report a case of Hirayama disease in a 25-year-old Indian man presenting with gradually progressive asymmetrical weakness and wasting of both hands and forearms along with unusual features of autonomic dysfunction and upper motor neuron lesion.

## 1. Introduction

Hirayama disease (HD), a rare neurological condition, is a sporadic juvenile muscular atrophy of the distal upper extremities, which predominantly affects the lower cervical cord. It mainly develops in the late teens and early twenties with a male preponderance. The typical clinical features include insidious onset and slow progression of unilateral or bilateral muscular atrophy with weakness of the forearms and hands. Sensory disturbance, autonomic involvement, and upper motor neuron (UMN) signs like hyperreflexia and hypertonia are rare [1]. The motor neuron disease (MND) is a very close differential diagnosis of HD, but, unlike MND, the disease progresses initially and is followed by spontaneous arrest several years after the onset. This disease is more prevalent in Japan and other Asian countries, but cases have been reported from other parts of the world as well.

## 2. Case Report

A 25-year-old Indian man presented with a 4 years' history of slowly progressive weakness and atrophy that started in the right hand and forearm. After one year, it progressed to involve the left hand as well. The hand weakness limited several activities of his daily living and he could no longer play cricket. He complained of tremulousness of both hands for the past one year that gradually progressed to involve both lower limbs within the next 6 months. The tremors resulted in severe disability in performing activities involving his hands like writing. He also developed excessive sweating of both palms. There was no history of neck pain, sensory involvement, difficulty in walking, dysphagia, diplopia, or bowel or bladder involvement. His past medical history was noncontributory; there was no trauma to the neck, exposure to toxins, or any allergies. None of his family members had a similar complaint.

**Figure 1:**
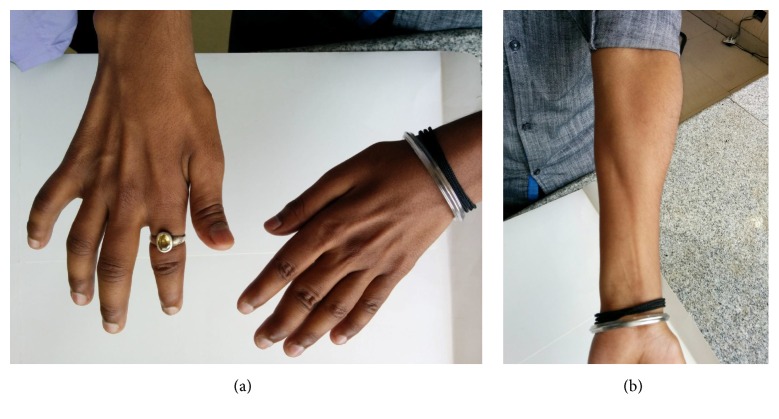
(a) Wasting of bilateral hands. (b) Wasting of the left forearm with sparing of brachioradialis muscle.

Neurological examination revealed features suggestive of both UMN and LMN lesions. Both forearms and hands were weak and wasted with preservation of brachioradialis muscles (Figures 1(a) and 1(b)). Full abduction, adduction of the digits, opposition of the thumbs, and palmar grasps were impaired. A coarse tremor was present in both hands and excessive sweating of palms was noted. There was no postural hypotension. Minipolymyoclonus was observed in bilateral quadriceps and calf muscles. There was hypertonia of both lower limbs and sustained bilateral ankle clonus was elicited. The Babinski sign was positive. The power of the proximal muscles of both upper and lower limbs was normal. There was no evidence of posterior column, cerebellum, or cranial nerve involvement.

Nerve conduction studies (NCS) showed reduced amplitude of compound muscle action potential (CMAP) in left median and ulnar nerve probably due to severe atrophy of the tested muscle. Sensory NCS was normal. Electromyography (EMG) revealed incomplete recruitment pattern with no evidence of positive sharp wave, fasciculation, fibrillation, and no spontaneous insertion activity. Amplitude of motor unit action potential was increased with slight increase in duration suggestive of neurogenic pattern. The autonomic sweat test was not performed due to nonavailability at the center.

Blood investigations including complete blood count, sedimentation rate, renal, liver, and thyroid function tests, creatine kinase, and vitamin B12 and vitamin D3 level were within normal range. There were negative results for vasculitis screening (rheumatoid factor, antinuclear antibody, extractable nuclear antigens, and antiphospholipid antibody) and viral serology: human immunodeficiency virus (HIV), hepatitis B, and hepatitis C.

**Figure 2:**
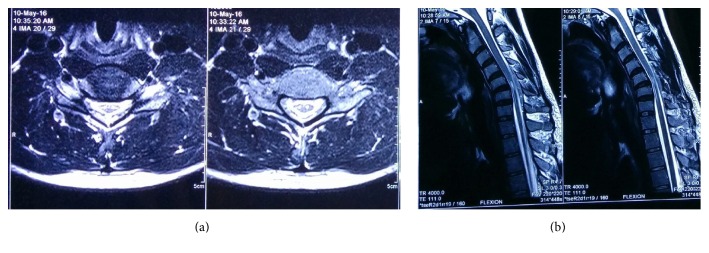
(a) MRI showing focal symmetrical atrophy of spinal cord with intramedullary T2 hyperintensity. (b) MRI showing forward displacement of the posterior subdural sac on flexion of neck, with prominent epidural space.

Multiplanar brain and cervical spine MRI was performed on 3-tesla magnet system using dedicated CP array head coils. Spin echo, turbo spin echo, and fluid attenuated inversion recovery (FLAIR) sequences were used to acquire T1 and T2 weighted images. Echo planar imaging was used to obtain diffusion weighted images and apparent diffusion coefficient maps. The MRI study (Figure 2(a)) revealed focal symmetrical atrophy of spinal cord in C5-C6 region with intramedullary T2 hyperintensity predominantly in the region of anterior horns. Flexion scan of the neck (Figure 2(b)) showed 5 mm forward displacement of posterior dural sac with prominent posterior epidural space appearing iso to hyperintense on T1 and T2 weighted image. The spinal cord was seen abutting the posterior margin of the vertebral body in that region. The straightening of cervical and lumbar spine was also noted. Scanning of the whole brain showed normal morphology and signal pattern.

Overall, the clinical, NCS, and MR imaging features were consistent with the diagnosis of Hirayama disease. The patient was prescribed a cervical collar to prevent neck flexion, thereby reducing further spinal cord injury.

## 3. Discussion

This disease was initially recognized in Japan in 1959 by Hirayama et al. and was reported under the name of “juvenile muscular atrophy of unilateral upper extremity” [2]. Although the disease is more prevalent in Asian countries like Japan, India, Sri Lanka, Singapore, Taiwan, and Hong Kong, similar cases have been reported across the world. The disease has also been described under various clinical entities in the literature as “juvenile muscular atrophy of the distal upper extremity, juvenile asymmetric segmental spinal muscular atrophy, and benign focal amyotrophy or monomelic amyotrophy” [3].

HD is characterized by insidious onset asymmetrical weakness and wasting of muscles of upper limb, affecting predominantly C7, C8, and T1 myotomes with male preponderance between 15 and 25 years of age. The disease usually progresses for few years (1–3) and then is followed by arrest of progression, rendering a relatively benign course. The clinical features may also be manifested as irregular coarse tremors (minipolymyoclonus) in the fingers of the affected hand(s) with mild transient worsening of symptoms on exposure to cold. The sensory, reflex, and cranial nerve examinations are generally normal. Pyramidal tract involvement in lower limb, autonomic disturbances, and cerebellar deficits are also rare. EMG of the affected muscles shows evidence of chronic denervation, with or without acute denervation changes (fasciculations, positive sharp waves, and fibrillations potentials). However, apparently healthy muscles may also show abnormal EMG findings [4].

Scarcity of the disease and several atypical reported cases pose a diagnostic challenge; Tashiro et al. [5] recently outlined the criteria requirements for diagnosis of HD:

1. Distal predominant muscle weakness and atrophy in forearm and hand
2. Involvement of the unilateral upper extremity almost always all the time
3. Onset between the ages of 10 to early 20s
4. Insidious onset with gradual progression for the first several years, followed by stabilization
5. No lower extremity involvement
6. No sensory disturbance and tendon reflex abnormalities
7. Exclusion of other diseases (e.g., motor neuron disease, multifocal motor neuropathy, brachial plexopathy, spinal cord tumors, syringomyelia, cervical vertebral abnormalities, anterior interosseous, or deep ulnar neuropathy)

Apart from these features, many authors [6] report sparing of brachioradialis muscle, giving the impression of an “oblique atrophy.”

Although the present patient met most of the criteria laid down by Tashiro et al., he also had excessive sweating of both palms (suggestive of autonomic dysfunction) and hypertonia of lower limbs with exaggeration of deep tendon reflexes with positive Babinski's sign (suggestive of UMN lesion). These autonomic and UMN lesions are rare presenting features of HD. Autonomic involvement has been reported in 36% and 46% of the cases in the series by Hassan et al. [7] and Gourie-Devi et al. [8], respectively. Similarly, UMN lesion has been observed in 18% and 12% of the cases reported by Hassan et al. [7] and Sonwalkar et al. [9], respectively.

The exact pathogenesis of HD is still unknown. A pathological study by Hirayama et al. [10] demonstrated cell shrinkage and necrosis, various degrees of degeneration of small and large nerve cells, mild gliosis, and some circulatory insufficiency in the anterior horns of the spinal cord from the lower cervical to upper thoracic levels, particularly at the C7 and C8 levels. Atopy and elevated serum IgE level have also been postulated to be precipitating factors in HD by some authors [11]. The most widely accepted hypothesis is a cervical myelopathy associated with neck flexion, proposed by Kikuchi et al. [12]. Normally, the spinal dura mater is loosely anchored to the vertebral canal by the nerve roots and the periosteum at the foramen magnum and the dorsal surfaces of C2 and C3 and the other at the coccyx. The relatively short and tight dura mater seen in patients with HD is unable to compensate for the increased length of the vertebral canal during neck flexion. This results in tightening of the dural canal during neck flexion, which leads to an anterior shift of the posterior dural wall, causing spinal cord compression against the vertebral body. This repeated neck flexion results in multiple episodes of ischaemia and chronic trauma to the spinal cord, which eventually leads to myelopathy, as evidenced by asymmetric lower cervical cord thinning in the MRI. Regarding the pathophysiology of UMN signs, different distribution of stress in the cervical cord was suggested by Kato et al. [13].

The differential diagnosis of HD includes the distal form of spinal muscular atrophy, amyotrophic lateral sclerosis (ALS), postpolio syndrome, multifocal motor neuropathy with conduction block, and toxic neuropathy as well as structural lesions of the cervical cord (syringomyelia). These clinical entities can be identified by their characteristic clinical, radiological, and electrophysiological features [2].

The key to diagnose this disease is based on the typical clinical features and dynamic MRI study when the neck is flexed. MR studies in flexion show not only the anterior displacement of the posterior wall but also a well-enhanced crescent-shaped lesion in the posterior epidural space of the lower cervical canal. This lesion typically disappears when the neck returns to a neutral position, confirming it to be a congested posterior internal vertebral venous plexus rather than a vascular malformation. MR imaging studies of the cervical spine in a neutral position can reveal several features such as localised lower cervical cord atrophy, asymmetrical cord flattening, and loss of attachment between the posterior dural sac and subjacent lamina, as well as noncompressed intramedullary high T2 signal intensity.

Hirayama disease is a self-limiting disorder and there is no consensus on the definitive treatment. However, early diagnosis is necessary because a cervical collar may arrest the progression of the disorder by limiting the neck flexion. Physiotherapy is also helpful in preventing complications resulting from immobility such as joint stiffness and muscle wasting [1].

In conclusion, we report a case of HD who presented with rare association of autonomic dysfunction and UMN signs. HD should always be considered in a young patient presenting with weakness and atrophic muscles of the hand and forearm.
